# Postpartum blues: a predictor of postpartum depression, from the IGEDEPP Cohort

**DOI:** 10.1192/j.eurpsy.2024.1741

**Published:** 2024-04-01

**Authors:** Alexandra Landman, Elodie Gaelle Ngameni, Marine Dubreucq, Julien Dubreucq, IGEDEPP Groups, Sarah Tebeka, Caroline Dubertret

**Affiliations:** 1 Department of Psychiatry, Louis-Mourier Hospital, AP-HP, Colombes, France; 2 Centre Referent de Rehabilitation Psychosociale, GCSMS REHACOOR 42, Saint-Étienne, France; 3 INSERM U1290 (Research on Healthcare Performance (RESHAPE)), University Lyon 1, Lyon, France; 4 Department of Child and Adolescent Psychiatry, University Hospital of Saint-Étienne, Saint-Priest-en-Jarez, France; 5 CNRS 5229 (Institute of Cognitive Neuroscience), University Lyon 1, Lyon, France; 6 Institute of Psychiatry and Neuroscience of Paris (IPNP), Université Paris Cité, INSERM U1266, Team 1, Paris 75014, France

**Keywords:** associated factors, IGEDEPP, postpartum, postpartum blues, postpartum depression, pregnancy

## Abstract

**Background:**

To identify the different factors associated with postpartum blues and its association with postpartum depression, from a large French cohort.

**Methods:**

We conducted an analysis of the Interaction Gene Environment in Postpartum Depression cohort, which is a prospective, multicenter cohort including 3310 women. Their personal (according to the Diagnostic and Statistical Manual, fifth edition [DSM-5]) and family psychiatric history, stressful life events during childhood, pregnancy, and delivery were collected. Likewise, the French version of the Maternity Blues Scale questionnaire was administered at the maternity department. Finally, these women were assessed at 8 weeks and 1 year postpartum by a clinician for postpartum depression according to DSM-5 criteria.

**Results:**

The prevalence of postpartum blues in this population was 33%, and significant factors associated with postpartum blues were found as personal (aOR = 1.2) and family psychiatric history (aOR = 1.2), childhood trauma (aOR = 1.3), obstetrical factors, or events related to the newborn, as well as an experience of stressful life events during pregnancy (aOR = 1.5). These factors had a cumulative effect, with each additional factor increasing the risk of postpartum blues by 31%. Furthermore, adjustment for sociodemographic measures and history of major depressive episode revealed a significant association between postpartum blues and postpartum depression, mainly at early onset, within 8 weeks after delivery (aOR = 2.1; 95% CI = 1.6–2.7), but also at late onset (aOR = 1.4; 95% CI = 1.1–1.9), and mainly if the postpartum blues is severe.

**Conclusion:**

These results justify raising awareness among women with postpartum blues, including reassurance and information about postpartum depression, its symptomatology, and the need for management in case of worsening or prolongation of postpartum blues.

## Introduction

First mentioned at the end of the 19th century, postpartum blues is a nonpathological manifestation to the hormonal upheaval that occurs in the days following childbirth, with a prevalence of 39% [1–3]. It usually lasts a few days, and includes depressive symptoms like sadness, crying, emotional lability, and fatigue that may be associated with insomnia, irritability, and anxiety [3–5].

Various factors have been found to be associated with postpartum blues, particularly personal psychiatric history. Bloch et al. found a significantly higher prevalence among women with psychiatric disorders related to hormonal changes that occur in a woman’s life (premenstrual dysphoric syndrome, mood changes at the time of puberty and during the third trimester of pregnancy) [6]. Moreover, a personal history of a major depressive episode was found to be associated with postpartum blues, with 20% of the history of a major depressive episode in women who experienced postpartum blues [6, 7]. Women with a history of postpartum depression (PPD) could also be at a greater risk of developing postpartum blues in a future pregnancy [8]. Furthermore, women with anxiety disorders also have a higher risk of postpartum blues [9]. Finally, a family psychiatric history, such as major depressive episodes in a first-degree relative, is also considered a factor associated with postpartum blues [7].

Obstetrical factors, such as primiparity; history of abortion; pregnancy complications (preeclampsia, fetal pathologies, or prenatal hospitalizations); or delivery (C-section) are also associated with postpartum blues [4, 8–11].

Sociodemographic and environmental factors should also be taken into account as they can play an important role in the life of a young mother. Although most studies do not find a link between age and postpartum blues, Hau et al. have drawn attention to a U-shaped curve with an increased risk of postpartum blues for women under 35 and over 39 years old [6, 7, 9, 12, 13]. In addition, Gerli et al. found an increased risk in women with a low level of education [8]. Socioeconomic insecurity is another notable associated factor, as is social isolation, or being a single mother and raising a child alone [1, 7, 11]. Life events and the environment in which a woman lives can also affect the period following childbirth. In addition, a partner suffering from anxiety disorders or family conflicts can also contribute to postpartum blues [9].

Although physiological, the postpartum blues can have harmful consequences on the mother as well as on the baby. As a matter of fact, women with postpartum blues may stop breastfeeding earlier and have reduced interactions with their newborns, which has adverse consequences for the health of the baby [14, 15]. Moreover, postpartum blues can develop into PPD, especially when symptoms are prolonged or very intense [13, 15–17]. PPD has harmful short- and long-term consequences for the mother, the child, and the mother–child relationship. These elements justify the importance of being able to prevent it and detect it as early as possible [15].

Although other studies have shown an association between postpartum blues and PPD, they often had bias and limitations, such as retrospective design for assessing the postpartum blues, the use of questionnaire to diagnose PPD, or small sample sizes. This study is, to our knowledge, the first of its kind to assess the role of postpartum blues on PPD, distinguishing it according to its time of onset.

The aim of the present study is to (i) identify the different factors associated with postpartum blues and (ii) evaluate its association with PPD from a large French cohort.

## Methods

### Participants

This study is based on the Interaction Gene Environment in Postpartum Depression (IGEDEPP) population, which is a prospective, multicenter cohort including 3310 women who gave birth in 8 different maternity units in Paris metropolitan area (France), between November 2011 and June 2016 [18]. To be included in the study, participants had to be older than 18, Caucasian, French-speaking, and covered by French social insurance. Women who gave birth before 32 weeks of gestation, had schizophrenia or mental retardation were excluded from the study.

The research protocol (ClinicalTrial.gov Identifier: NCT01648816), including informed consent procedures, was approved by the French Ethics Committee (Île-de-France I) and Data Protection and Freedom of Information Commissions.

### Measures

Women were assessed by a trained psychiatrist or psychologist. The assessment took place at three different times during the year following their delivery. The first interview took place in a face-to-face format at the maternity hospital between the second and the fifth day after delivery. The second and third interviews were conducted by phone at 8 weeks and 1 year postpartum.

### Screening and diagnostic tools for postpartum blues and depression

To assess postpartum blues and its intensity, we used the validated French version of the Maternity Blues Scale by Kennerley and Gath, one of the 2 widely used scales [1]. It is a 28-item self-questionnaire that identifies seven different groups according to the score: primary blues, reservation, hypersensitivity, depression, despondency, retardation, and decreased self-confidence [19, 20]. It was administered to women between the second and the fifth day after delivery [19]. A score ≥7, out of a total of 28, identifies postpartum blues [20]. In our study, 3304 completed this questionnaire.

Regarding PPD, it was assessed in all women using the Diagnostic Interview for Genetic Studies (DIGS), a semi-structured interview, at three distinct time points: at inclusion, for the diagnosis of current and past depressive episodes; at 8 weeks after delivery, for the diagnosis of early-onset PPD; and at 1 year, for late-onset PPD [21]. Late-onset PPD was defined as a major depressive episode occurring between 2 months and 1 year after delivery and was, retrospectively, assessed by asking women about the presence and duration of depressive symptoms during the previous 10 months. All diagnoses of PPD were based on the Diagnostic and Statistical Manual, fifth edition (DSM-5) criteria for major depressive episodes [22]. Women assessed at all three time points, with no early- or late-onset PPD, will constitute our control group.

#### Sociodemographic measures and stressful events in childhood

Sociodemographic measures were assessed during the first interview at the maternity department. These included age, marital status, education, and employment. Several age groups were combined: (i) 18–25, (ii) 26–34, (iii) 35–39, and (iv) 40 and older. Marital status was assessed using six categories that were then combined to form two groups: (i) widowed, divorced, separated, never married and (ii) married or cohabiting. Education level was divided into two levels: (i) high school or less and (ii) university or more. Regarding employment, women were classified as (i) employed and (ii) unemployed.

Childhood trauma was investigated using a self-administered questionnaire, the Childhood Trauma Questionnaire, consisting of 28 questions addressing five types of childhood trauma: sexual abuse, physical or emotional neglect and abuse [23]. Threshold scores were defined by Paquette et al. to determine the presence of abuse and/or neglect: >10 for physical or sexual abuse, >13 for physical neglect, and >15 for emotional abuse or neglect [24].

#### Lifetime personnel and family psychiatric history

The psychiatric history of the women included in the study was evaluated by a psychologist or psychiatrist, using the DIGS, according to DSM-5 criteria [21, 22, 25]. Thus, the lifetime prevalence of mood disorders (major depressive episode and bipolar disorder); anxiety disorders (panic disorder, social phobia, specific phobia, generalized anxiety disorder, obsessive–compulsive disorder, and agoraphobia); and substance use disorders (tobacco, alcohol, cannabis, cocaine, opiates, stimulants, and other drugs) was estimated, as well as eating disorders (anorexia and bulimia). The history of suicide attempts was also assessed.

Regarding family history of psychiatric disorders, the Family Informant Schedule and Criteria was used to assess mood disorders, anxiety disorders, schizophrenia, and addictions [26]. The presence of one of these disorders in a first-degree relative thus allowed us to consider a family psychiatric history in these patients.

#### Obstetrical factors and stressful events during pregnancy

Obstetrical characteristics were collected at the maternity department using a hetero questionnaire to assess the following: previous pregnancies, infertility before this pregnancy, use of assisted reproductive technology, existence of chronic diseases, history of multiple pregnancies, emergency room consultations, and hospitalizations during pregnancy (for hypertension, threat of preterm delivery, gestational diabetes, thromboembolic event). Data concerning delivery included labor onset (spontaneous or induced), mode of delivery (vaginal or cesarean), use of obstetrical analgesia (yes or no), and perineal trauma (no, or trauma if the tearing degree is ≥2). Prematurity (<37 weeks of gestation), low birth weight (<2500 g), presence of postpartum hemorrhage, need for intensive care or resuscitation for the mother or the newborn, and breastfeeding mode (breast-feeding or bottle-feeding) were also considered.

To identify stressful events during pregnancy, the Paykel scale was used; it is a 64-item questionnaire that measures the subjective impact of each event as well as the consequences and life changes, which it caused. These items are divided into categories: work, education, health, bereavement, romantic relationships, legal proceedings, relocation, familial, and marital status. Life events were considered stressful when participants rated them as having a “strong” or “severe” negative impact (impact score of 1 or 2) [27]. Finally, stressful events were defined by the subjective experience of the event, not by its objective severity. The Paykel scale was used in the maternity department to assess stressful events during pregnancy.

### Statistical analysis

We conducted an analysis of the IGEDEPP cohort. We identified women who experienced postpartum blues, defined as having a score ≥7 on the Maternity Blues Scale between the second and the fifth day after delivery.

Descriptive statistics for categorical variables included numbers and percentages for each category and by case/control status. Multivariate logistic regressions were performed to quantify the association between postpartum blues and associated factors. Odds ratios (ORs) adjusted for sociodemographic variables (i.e., age, marital status, education, and employment), are reported with their 95% confidence intervals (95% CI) and *p* values. The significance level was set at 0.05.

Sociodemographic variables and variables with *p* values less than 0.2 in the first analysis were included as adjustment factors in the multivariate model, using a stepwise descending analysis of the variables.

Finally, to assess the association between postpartum blues and PPD, we estimated ORs and their 95% CIs using logistic regressions. We present the results of (i) univariate and multivariate analyses adjusted for (ii) sociodemographic variables (age, marital status, education level, unemployment) and (iii) for the same sociodemographic variables and a lifetime history of major depressive episode.

All analyses were performed with R, v3.6.1.

## Results

At the maternity department, 3310 women were included (for more details on the IGEDEPP cohort description, see Tebeka et al. [28]). Among them, 3304 completed the Maternity Blues Scale and our work focused on these women.

### Prevalence of postpartum blues and severity

The prevalence of postpartum blues was found to be 33.0% (*N* = 1091) in the overall population.

The mean score of the Maternity Blues Scale was 5.57 (median 5.0, first interquartile range 2, third interquartile range 8, and maximum score 23).

### Sociodemographic data

The sociodemographic characteristics of each of the two groups are summarized in Table 1. Majority of women were between 25 and 40 years old (87.7%), with a mean age of 32. Most participants were married or in domestic partnerships (96.9%), employed (93.3%), and had a high level of education (92.1%). No significant differences in sociodemographic data were found between women who experienced postpartum blues and those who did not (Table 1).

**Table 1.** Association between postpartum blues and sociodemographic data and childhood trauma (CTQ)

	Postpartum blues (*N* = 1091)	Controls (*N* = 2213)	Postpartum blues vs. controls	
	*N* (%)	*N* (%)	aOR (95% CI)	*p*
Age (years)				
<25	88 (8.1)	156 (7.0)	1.15 (0.87–1.51)	0.5168
Between 25 and 40	953 (87.4)	1946 (87.9)	Ref	
>40	50 (4.6)	111 (5.0)	0.92 (0.65–1.2)	
Marital status: single	43 (3.9)	60 (2.7)	1.5 (1.0–2.2)	0.0571
Education level: primary or high school	100 (9.2)	161 (7.3)	1.3 (1.0–1.7)	0.0586
Unemployed	84 (7.7)	138 (6.2)	1.3 (0.9–1.7)	0.115
**Childhood stressful life event** (CTQ, *N* above threshold)				
Any trauma	124 (11.6)	174 (8.0)	**1.5 (1.1–1.9)**	**0.00218**
Emotional abuse	42 (3.9)	58 (2.7)	1.5 (1.0–2.2)	0.063
Physical abuse	31 (2.9)	40 (1.8)	1.5 (0.9–2.4)	0.0944
Sexual abuse	39 (3.7)	47 (2.2)	**1.7 (1.1–2.6)**	**0.0188**
Emotional neglect	86 (8.1)	110 (5.0)	**1.6 (1.2–2.1)**	**0.00229**
Physical neglect	15 (1.4)	11 (0.5)	**2.6 (1.2–5.7)**	**0.0179**

*Note*: Significant results are represented in bold.

Abbreviations: aOR, adjusted OR for age, marital status, education, and employment; CTQ, childhood trauma questionnaire; OR, odds ratio.

### Traumatic events during childhood

Women who experienced childhood trauma of any kind (aOR = 1.5; 95% CI = 1.1–1.9; *p* = 0.002), particularly sexual abuse (aOR = 1.7; 95% CI = 1.1–2.6; *p* = 0.02), emotional neglect (aOR = 1.6; 95% CI = 1.2–2.1; *p* = 0.002), and physical neglect (aOR = 2.6; 95% CI = 1.2–5.7; *p* = 0.02) had a higher risk of developing postpartum blues, after adjusting for age, marital status, education, and employment (Table 1). No significant association was found for women who were physically or emotionally abused.

### Personal lifetime and family psychiatric history

Regarding personal psychiatric history, after adjustment for age, marital status, education level, and employment, an increased risk of postpartum blues was found, with an aOR of 1.5 (95% CI = 1.3–1.7; *p* < 0.001) in women with any psychiatric disorder history (Table 2). More specifically, a history of a major depressive episode (aOR = 1.4; 95% CI = 1.2–1.6; *p* < 0.001), anxiety disorders (aOR = 1.6; 95% CI = 1.4–2.0; *p* < 0.001), or tobacco dependence (aOR = 1.3; 95% CI = 1.0–1.8; *p* = 0.04) were associated factors. However, a history of suicide attempts, eating disorders, and substance use disorders were not found to be significant associated factors.

**Table 2.** Association between postpartum blues and personal and family psychiatric history

	Postpartum blues (*N* = 1091)	Controls (*N* = 2213)	Postpartum blues vs. controls	
	*N* (%)	*N* (%)	aOR (95% CI)	*N* (%)
**Personal psychiatric history**				
Any psychiatric disease	598 (54.8)	985 (44.5)	**1.5 (1.3–1.7)**	**<0.001**
Major depressive episode	443 (40.6)	722 (32.6)	**1.4 (1.2–1.6)**	**<0.001**
Suicide attempt	42 (3.8)	56 (2.5)	1.5 (1.0–2.2)	0.0761
Any anxiety disorder	234 (21.4)	312 (14.1)	**1.6 (1.4–2.0)**	**<0.001**
Any eating disorder	45 (4.1)	92 (4.2)	1.0 (0.7–1.4)	0.981
Any substance use disorder	104 (9.5)	176 (8.0)	1.2 (0.9–1.5)	0.238
Tobacco dependence	94 (8.6)	141 (6.4)	**1.3 (1.0–1.8)**	**0.0402**
Alcohol use disorder	10 (0.9)	10 (0.5)	1.9 (0.8–4.6)	0.159
Cannabis use disorder	17 (1.6)	38 (1.7)	0.9 (0.5–1.5)	0.599
**Family psychiatric history**				
Any psychiatric disorder	754 (69.1)	1387 (62.7)	**1.3 (1.1–1.6)**	**<0.001**
Mood disorder	585 (53.6)	1039 (46.9)	**1.3 (1.1–1.5)**	**<0.001**
Anxiety disorder	272 (24.9)	424 (19.2)	**1.4 (1.2–1.7)**	**<0.001**
Schizophrenia	17 (1.6)	30 (1.4)	1.2 (0.6–2.1)	0.648
Alcohol dependence or abuse	167 (15.3)	334 (15.1)	1.0 (0.8–1.2)	0.924
Other substance use disorder	232 (21.3)	415 (18.8)	1.2 (1.0–1.4)	0.0965

*Note*: Significant results are represented in bold.

Abbreviations: aOR, adjusted OR for age, marital status, education, and employment; OR, odds ratio.

Women with a family psychiatric history also had an increased risk of postpartum blues after adjustment for age, marital status, education level and employment (aOR = 1.3; 95% CI = 1.1–1.6; *p* < 0.001). Especially, mood disorder (aOR = 1.3; 95% CI = 1.1–1.5; *p* < 0.001) and anxiety disorder (aOR = 1.4; 95% CI = 1.2–1.7; *p* < 0.001) in a first-degree relative significantly increased this risk (Table 2).

### Obstetrical factors and stressful events during pregnancy

A higher risk of postpartum blues was found in women who had experienced at least one stressful event with negative psychological impact during their pregnancy according to the Paykel scale, with an aOR of 1.5 (95% CI = 1.3–1.8; *p* < 0.001) (Table 3).

**Table 3.** Association between postpartum blues and stressful events during pregnancy and obstetrical factors

	Postpartum blues (*N* = 1091)	Controls (*N* = 2213)	Postpartum blues vs. controls	
	*N* (%)	*N* (%)	aOR (95% CI)	*p*
**Stressful events during pregnancy**				
At least one stressful event with negative impact during **pregnancy (Paykel scale)**	601 (55.1)	977 (44.1)	**1.5 (1.3–1.8)**	**<0.001**
**Obstetrical events before and during pregnancy**				
Primiparity	707 (64.8)	1189 (53.7)	**1.6 (1.4–1.9)**	**<0.001**
Infertility	149 (13.7)	251 (11.3)	**1.3 (1.0–1.6)**	**0.0287**
Assisted reproductive technology	101 (9.3)	167 (7.5)	**1.3 (1.0–1.7)**	**0.0501**
Physical concomitant chronic disease	162 (14.9)	289 (13.1)	1.2 (0.9–1.4)	0.165
Multiple pregnancy	46 (4.2)	59 (2.7)	**1.6 (1.1–2.4)**	**0.0138**
Emergency consultation during pregnancy	563 (51.7)	1030 (46.5)	**1.2 (1.1–1.4)**	**0.00614**
Hospitalization during pregnancy	166 (15.2)	273 (12.3)	**1.3 (1.0–1.6)**	**0.0269**
Threatened preterm labor	49 (29.5)	90 (33.0)	0.8 (0.6–1.3)	0.442
Hypertension during pregnancy	17 (10.2)	20 (7.3)	1.5 (0.8–3.0)	0.253
Gestational diabetes	17 (10.2)	42 (15.4)	0.6 (0.3–1.2)	0.151
Venous thromboembolic event	3 (1.8)	4 (1.5)	1.2 (0.3–5.4)	0.846
**Delivery events**				
Non–spontaneous labor	509 (46.7)	812 (36.7)	**1.5 (1.3–1.8)**	**<0.001**
C–section delivery	324 (29.7)	493 (22.3)	**1.5 (1.3–1.7)**	**<0.001**
No obstetrical analgesia despite intention	177 (16.2)	339 (15.32)	1.4 (0.8–2.6)	0.28
Perineal trauma (NA = 824)	573 (74.8)	1116 (64.9)	**1.6 (1.4–2.0)**	**<0.001**
Newborn related events (preterm, small for gestational age, NICU)	133 (12.2)	177 (8.0)	**1.6 (1.3–2.0)**	**<0.001**
Early maternal postpartum events (hemorrhage, ICU)	68 (6.2)	112 (5.1)	1.3 (0.9–1.7)	0.153

*Note*: Significant results are shown in bold.

Abbreviations: aOR, adjusted OR for age, marital status, education, and employment; ICU, intensive care unit; NICU, neonatal intensive care unit; OR, odds ratio.

After adjustment for age, marital status, education, and employment, a higher risk of postpartum blues was found in primiparous women (aOR = 1.6; 95% CI = 1.4–1.9; *p* < 0.001), those with infertility (aOR = 1.3; 95% CI = 1.0–1.6; *p* = 0.03), or who had undergone assisted reproductive technology (aOR = 1.3; 95% CI = 1.0–1.7; *p* = 0.05), or in those with multiple pregnancies (aOR = 1.6; 95% CI = 1.1–2.4; *p* = 0.01) (Table 3). In addition, women who had consulted the emergency room (aOR = 1.2; 95% CI = 1.1–1.4; *p* = 0.006) or who had been hospitalized during their pregnancy (aOR = 1.3; 95% CI = 1.0–1.6; *p* = 0.03), also had an increased risk of postpartum blues.

Concerning delivery; induced labor (aOR = 1.5; 95% CI = 1.3–1.8; *p* < 0.001); cesarean section (aOR = 1.5; 95% CI = 1.3–1.7; *p* < 0.001); perineal trauma (aOR = 1.6; 95% CI = 1.4–2.0; *p* < 0.001); and newborn events (prematurity, low weight for gestational age, or need for intensive care) (aOR = 1. 6; 95% CI = 1.3–2.0; *p* < 0.001) were found to be associated factors, whereas the absence of epidural and early maternal postpartum events (delivery hemorrhage or need for intensive care) did not show a significant increase in risk.

### Factors associated with postpartum blues: Multivariate analysis

After multivariate analysis, the factors independently associated with postpartum blues were childhood trauma (aOR = 1.3; 95% CI = 1.0–1.7; *p* < 0.001), a stressful event with negative impact during pregnancy (aOR = 1. 6; 95% CI = 1.3–1.8; *p* < 0.001), primiparity (aOR = 1.6; 95% CI = 1.4–1.9; *p* < 0.001), multiple pregnancies (aOR = 1.6; 95% CI = 1.0–2.5; *p* = 0.03), induced labor (aOR = 1.4; 95% CI = 1.2–1.6; *p* < 0. 001), perineal trauma (aOR = 1.6; 95% CI = 1.3–1.9; *p* < 0.001), newborn events (aOR = 1.4; 95% CI = 1.0–1.8; *p* = 0. 03), history of depressive episode (aOR = 1.2; 95% CI = 1.0–1.4; *p* = 0.02), anxiety disorders (aOR = 1.5; 95% CI = 1.2–1.8; *p* < 0.001), and family history of anxiety disorders (aOR = 1.2; 95% CI = 1.0–1.5; *p* = 0.02) (Table 4).

**Table 4.** Multivariate logistic regression model for postpartum blues

	Postpartum blues vs. controls	
	aOR (95% CI)	*p*
Chlildhood trauma (CTQ)	**1.3 (1.0–1.7)**	**<.001**
Stressor event with negative impact during pregnancy (Paykel)	**1.6 (1.3–1.8)**	**<.001**
Primiparity	**1.6 (1.4–1.9)**	**<.001**
Multiple pregnancy	**1.6 (1.0–2.5)**	**0.034**
Non–spontaneous labor	**1.4 (1.2–1.6)**	**<.001**
Perineal trauma	**1.6 (1.3–1.9)**	**<.001**
Newborn related events (preterm, small for gestational age, NICU)	**1.4 (1.0–1.8)**	**0.030**
Personal depressive disorder	**1.2 (1.0–1.4)**	**0.018**
Personal anxiety disorder	**1.5 (1.2–1.8)**	**<.001**
Family history of anxiety disorder	**1.2 (1.0–1.5)**	**0.021**

*Note*: Significant results are shown in bold.

Abbreviations: aOR, adjusted OR; CTQ, childhood trauma questionnaire.

Women with postpartum blues had an average of 3.4 of these factors (vs. 2.7 in the control group, *p* ≤ 0.001). The number of this factor was also associated with an increased risk of postpartum blues per additional factor (aOR = 1.3, 95% CI = 1.00–1.5, *p* ≤ .001).

### Association between postpartum blues and PPD

**Figure 1.**
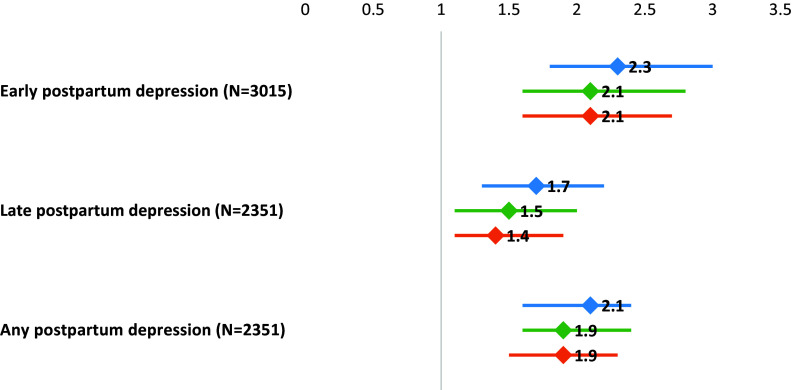
Forest plot showing the association between postpartum blues and postpartum depression in women assessed at 1 year postpartum.

Among women with postpartum blues, 27.7% experienced either early- or late-onset PPD, compared to 16.4% among women without postpartum blues (OR = 2.0; 95% CI = 1.6–2.4) (Figure 1). More specifically, 15.5% had early-onset PPD versus 7.8% for those without postpartum blues (OR = 2.3; 95% CI = 1.8–3.0), and 12.6% had late-onset PPD, versus 8.7% for those without postpartum blues (OR = 1.7; 95% CI = 1.3–2.2).

After adjustment for sociodemographic data, the increased risk was confirmed. Indeed, an aOR of 2.1 (95% CI = 1.6–2.8) was found for early-onset PPD, of 1.5 (95% CI = 1.1–2.0) for late-onset PPD and of 1.9 (95% CI = 1.6–2.4) for all PPD.

Finally, after adjustment on the sociodemographic data and the history of major depressive episode, concordant results were found: aOR of 2.1 (95% CI = 1.6–2.7) for early-onset PPD, aOR of 1.4 (95% CI = 1.1–1.9) for late-onset PPD, and aOR of 1.9 (95% CI = 1.5–2.3) for any PPD (Figure 1).

**Figure 2.**
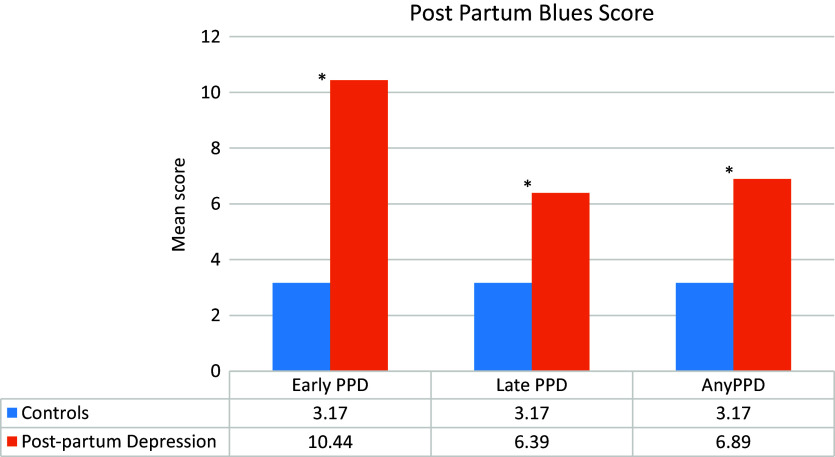
Mean postpartum blues score by early and late postpartum depression.

In addition, a mean Maternity Blues Scale score of 6.89 was found in women with PPD (10.44 for women with early-onset PPD and 6.39 for those with late-onset PPD), versus 3.17 in women with no PPD (*p* < 0.001) (Figure 2).

## Discussion

In our study, postpartum blues concerned 33.0% of women. In our most comprehensive multivariate model, postpartum blues was significantly associated with numerous factors such as childhood trauma, stressor events with negative impact during pregnancy, primiparity, multiple pregnancy, induced labor, perineal trauma, newborn related events, personal history of depressive disorder, and personal or family history of anxiety disorder. These factors had a cumulative effect: each added 31% to the risk of developing postpartum blues. Furthermore, we found a significant association between postpartum blues and PPD, mainly at early onset, within 8 weeks of delivery but also at late-onset, and particularly if the postpartum blues was severe.

The postpartum blues prevalence of 33.0% in the IGEDEPP cohort corresponds to the literature’s data. Indeed, in a large meta-analysis of 26 international studies, 7 European studies used the Maternity Blues Scale, with a pooled prevalence of postpartum blues of 39.3% (95% CI = 26.7–52.0) [1]. In our study, the rate is slightly lower, but remains within the same range. A generally advantaged Caucasian population due to the initial study design (IGEDEPP’s main objective being a genetic analysis requiring ethnic homogeneity) can explain this difference [28].

Concerning associated factor, experienced childhood trauma was associated with an increased risk of postpartum blues, particularly sexual abuse, emotional neglect or physical neglect. Interestingly, the literature did not provide any insight between childhood trauma and postpartum blues. It could be explained by the fact that, as this is not a pathological condition, the studies usually focus on psychiatric and obstetric conditions and their possible link with postpartum blues. In contrast, many studies found an association between childhood trauma and PPD [29–32].

Our work, like others before it, emphasizes the importance of personal and family psychiatric history and obstetrical factors in the postpartum blues [1, 4, 6–9, 13]. We found a close association between personal psychiatric history and postpartum blues, specifically with a history of major depressive episode and anxiety disorders, confirmed after multivariate analysis, which are also found as associated factors by several studies. O’Hara et al. [7] found a 61.7% history of depressive episodes in women with postpartum blues, compared with 28.4% in those without blues. Bloch et al. [6] reported 20% of depressive episodes in the history of women with postpartum blues, compared with 3% in those without. Finally, Luciano et al. [9] found an increased risk of postpartum blues in women with a history of anxiety disorders, with an OR of 3.16. Some family psychiatric histories were also found to be significant associated factors with postpartum blues in our study, in particular mood disorders and anxiety disorders. O’Hara et al. [7] also found this link, with 57.4% of family history of depressive episodes in women with postpartum blues, compared with 31.3% in those without postpartum blues. After multivariate analysis, we found that only family history of anxiety disorders was associated with postpartum blues. To our knowledge, this association has never been shown.

Regarding the impact of stressful life events during pregnancy and obstetrical factors, our study identified several significant associated factors with postpartum blues. These factors included the occurrence of at least one stressful event during pregnancy with a negative impact, being a primiparous mother, experiencing infertility and utilizing assisted reproductive technology, having a multiple pregnancy, requiring emergency consultation or hospitalization. Additionally, induced labor, cesarean delivery, perineal trauma, and events related to the newborn after delivery were found to be significant factors associated with postpartum blues. Stressful events with negative impact occurring during pregnancy; obstetrical factors (primiparity, multiple pregnancies, induced labor, perineal trauma); and events related to the newborn after delivery were still associated with postpartum blues after multivariate analysis. Similar results were also found in other studies. Ntaouti et al. [4] reported an increased risk of postpartum blues in primiparous women. Adewuya et al. and Luciano et al. noted that obstetrical or fetal complications during pregnancy induced an increased risk of postpartum blues [9, 11]. O’Hara et al. [7] noted an increased risk of postpartum blues after an event with a negative impact during pregnancy, or in women whose baby had complications at birth, but did not find any link concerning childbirth. Nevertheless, Gerli et al. [8] reported that the course of childbirth, including anxiety, complications and the need for a cesarean section, had an influence on the risk of postpartum blues. We did not find any previous data concerning perineal trauma. An explanation could be that the pain associated with perineal trauma is on the causal pathway to postpartum blues [33, 34]. Luciano et al. [9], contrary to our study, did not find a link between postpartum blues and the need for intensive care of the newborn.

We have shown a clear association between postpartum blues and PPD, whether the onset is early or late, even after adjustment for sociodemographic characteristics and personal history of major depressive episode. In addition, we found significantly higher mean postpartum blues scores, reflecting the severity of postpartum blues symptoms, in women who developed PPD secondarily, underlining that severe postpartum blues appeared to be associated with a higher risk of progression to PPD. Several studies have found a link between postpartum blues and PPD, as well as the influence of the severity of postpartum blues on the development of PPD [6, 13, 15–17, 35]. This association between blues and PPD may possibly be explained by the fact that women with postpartum blues and those with PPD share many characteristics. Indeed, Tebeka et al., distinguishing early-onset PPD from late-onset depression, found many risk factors for early PPD that we also found in our study as factors associated with postpartum blues, such as personal psychiatric history, specifically major depressive episodes, anxiety disorders, tobacco dependence, family psychiatric history of mood disorders, history of sexual abuse, emotional neglect or any childhood trauma, stressful events with negative impact, or emergency visits during pregnancy. In their study, they found an association between being single, having a low level of education, having a history of emotional abuse in childhood, having attempted suicide, having a substance use disorder, or having a concurrent chronic illness and early PPD. However, we did not find an association between these elements and postpartum blues. In addition, we found an association between postpartum blues and a history of physical neglect, primiparity, infertility, use of assisted reproduction, and multiple pregnancies, whereas this study did not find an association between these elements and PPD [28]. These elements suggest that the factors associated with postpartum blues partially overlap with those associated with early-onset PPD, thus highlighting a certain specificity of these manifestations.

Our study allowed us to underline the association between postpartum blues and late-onset PPD after adjustment for sociodemographic measures and history of major depressive episode (aOR = 1.4; 95% CI = 1.1–1.9). This original finding, not found in previous study, further clarifies that postpartum blues is a risk factor for PPD, independently of a simple continuity. If postpartum blues is a physiological and transient condition and does not require treatment, according to international recommendations, assessing the risk of developing PPD seems necessary [4, 36].

These findings support the importance of early screening and awareness of women experiencing postpartum blues. Health professionals should be aware that postpartum blues can lead to PPD as it is crucial to provide these women with reassurance, but also information about PPD, its symptoms, and the significance of seeking appropriate medical attention if their symptoms worsen or persist. Such measures can potentially improve the long-term outcomes of the mother–baby relationships but also have an impact on the incidence of PPD.

Our study has several strengths. First, the sample size; indeed, IGEDEPP is the largest prospective multicenter study to assess PPD at three distinct time points in the year after delivery. Second, an important element is the diagnosis of PPD that was made by a clinician, based on DSM-5 criteria, not using only questionnaires. Furthermore, to the best of our knowledge, no study has investigated the role of postpartum blues on PPD, distinguishing it according to its time of onset. Finally, the exhaustive evaluation of the women’s personal and family psychiatric history, traumatic life events experienced in childhood, stressful events experienced during pregnancy and obstetrical events, allows for a complete analysis of the women’s profile.

Regarding the main limitation of the study, most of the women included in the sample had favorable sociodemographic characteristics, with a high level of education, a job, and lived in cohabitation, which is not representative of the French population and may thus limit the external validity of our study. Finally, the Maternity Blues Scale was filled out between days 2 and 5, which may cause a change in our assessment of the postpartum blues.

## Conclusion

We find that postpartum blues and its severity are associated with PPD, regardless of the onset timing. This can be explained by the fact that several associated factors are common to both entities. Future research should explore the effects of early awareness and intervention specifically in cases of severe postpartum blues. Furthermore, prospective studies should focus on a particularly vulnerable subpopulation, as they accumulate the factors identified; this could lead to a real improvement in the postpartum mental health.

## Data Availability

The data that support the findings of this study are available from APHP. Restrictions apply to the availability of these data, which were used under license for this study. Data are available from the authors with the permission of APHP.
